# Comparative validation of oxidative bisulfite sequencing (oxBS) and chemical-assisted pyridine borane sequencing (CAPS) protocols for locus-specific 5-hydroxymethylcytosine quantification

**DOI:** 10.1186/s13072-026-00672-3

**Published:** 2026-04-20

**Authors:** Katharina Pühringer, Philipp Czarda, Sebastian Iluca, Benno Fehringer, Pece Sherovski, Angelica Ohindovschi, Andreas Hainfellner, Lukas Reissig, Wolfgang J. Weninger, Margit Cichna-Markl

**Affiliations:** 1 Faculty of Chemistry, Institute of Analytical Chemistry, University of Vienna, Vienna, Austria; 2 Vienna Doctoral School in Chemistry (DoSChem), University of Vienna, Vienna, Austria; 3 Institute of Chemistry, Faculty of Natural Sciences and Mathematics, Ss. Cyril and Methodius University in Skopje, Skopje, North Macedonia; 4 Department of Pharmacognosy and Pharmaceutical Botany, Nicolae Testemițanu State University of Medicine and Pharmacy, Chișinău, Republic of Moldova; 5 Division of Anatomy, Centre for Anatomy and Cell Biology, Medical University of Vienna, Vienna, Austria

**Keywords:** 5-hydroxymethylcytosine, DNA methylation, Bisulfite sequencing (BS), Oxidative bisulfite sequencing (oxBS), Chemical-assisted pyridine borane sequencing (CAPS), Method validation

## Abstract

**Background:**

DNA methylation is a key regulator of tissue-specific gene expression, cell differentiation, and development. In mammals, DNA methylation predominantly occurs as 5-methylcytosine (5mC) at CpG dinucleotides. DNA methylation is a dynamic and reversible process. Increasing evidence indicates that 5-hydroxymethylcytosine (5hmC), an oxidation product of 5mC, plays important roles in chromatin organization and gene regulation. Aberrant 5hmC levels have been associated with various neurological disorders and cancer types. Despite its biological relevance, accurate quantification of 5hmC at single-CpG resolution remains challenging due to its low abundance and methodological limitations of existing detection approaches.

**Results:**

Here, we systematically compared bisulfite-based, bisulfite-free, and hybrid DNA conversion strategies for 5hmC analysis at single-CpG resolution with respect to accuracy, repeatability, lowest detectable (LDL) and lowest quantifiable (LQL) 5hmC levels, and DNA recovery. Method performance was evaluated using a 97 nt fragment of an *MGMT* enhancer containing two CpG sites. Synthetic oligonucleotides carrying cytosine, 5mC, or 5hmC at variable positions revealed that chemical-assisted pyridine borane sequencing (CAPS), a bisulfite-free approach, yielded the most accurate 5hmC quantification. However, false-positive signals resulted in increased LDL and LQL values, limiting its applicability to samples with low 5hmC abundance. Bisulfite-based protocols generally showed higher sensitivity, with the LDL and LQL values depending on the total incubation time. An optimized oxidative bisulfite protocol with a total incubation time of 140 min achieved low LDL and LQL values while substantially reducing the incubation time compared to previously published conditions. Among the oxidants tested, K_2_RuO_4_ resulted in more repeatable results than KRuO_4_. Bisulfite-based protocols showed superior DNA recovery compared to bisulfite-free ones.

**Conclusion:**

Our results demonstrate that bisulfite-based and bisulfite-free approaches for 5hmC analysis at single-CpG resolution exhibit distinct analytical strengths and limitations. Method selection should therefore be based on the specific biological question, the expected abundance of 5hmC, and practical constraints, such as DNA input and sample integrity, rather than by accuracy or sensitivity alone. It should be noted that analytical validation was primarily performed using single-stranded oligonucleotides.

**Supplementary Information:**

The online version contains supplementary material available at 10.1186/s13072-026-00672-3.

## Background

DNA methylation is an epigenetic mark that plays a key role in tissue-specific gene expression, cell differentiation, and development [1]. Aberrant DNA methylation has been associated with various diseases including cancer [2, 3]. In mammals, DNA methylation predominantly occurs at the 5-position of cytosine (5mC) within CpG dinucleotides. Although once considered a relatively stable modification, DNA methylation is now recognized as a dynamic process [4]. It is established and maintained by DNA methyltransferases [5], while DNA demethylation can occur by both active and passive mechanisms.

The oxidation of 5mC to 5-hydroxymethylcytosine (5hmC) by ten-eleven translocation enzymes is the initial step of active DNA demethylation [6, 7]. Growing evidence suggests that 5hmC is not only a transient intermediate, but also a stable [8] and functionally relevant epigenetic mark that has distinct regulatory properties and interacts with other epigenetic mechanisms [9–12]. It is particularly abundant in the central nervous system, where it appears to play an important role in neural development and function [13, 14]. Altered 5hmC levels have been linked to several neurological disorders, including Alzheimer’s disease [15] and Parkinson’s disease [16], as well as several cancer types such as melanoma [17], colorectal and gastric cancers [18], and brain tumors [19]. Due to its biochemical stability, 5hmC has emerged as a promising diagnostic and prognostic biomarker in cancer [20].

Global 5hmC levels can be determined by various methodologies, including HPLC-MS/MS [21], antibody-based techniques [22], and more recent approaches such as nanoparticle-based biosensors [23]. Due to the relatively low abundance of 5hmC in the genome, its analysis at single-CpG resolution is technically more demanding [24]. For site-specific 5hmC detection, both bisulfite-based and bisulfite-free methods have been developed. Bisulfite-based methods rely on the selective conversion of unmethylated cytosines to uracil within single-stranded DNA (ssDNA) [25–27]. Since both 5mC and 5hmC remain unconverted, an additional reaction is required to differentiate between these two modifications. In this second reaction, bisulfite treatment is preceded by an oxidation step converting 5hmC to 5-formylcytosine (5fC), which is susceptible to bisulfite conversion [28, 29]. 5hmC levels can then be calculated by subtracting values from the oxidative bisulfite treatment (reflecting 5mC only) from those obtained after conventional bisulfite conversion (reflecting 5mC + 5hmC) [28, 29]. However, deriving 5hmC levels from the difference of two measurements leads to error propagation and consequently increases the overall measurement uncertainty. In addition, bisulfite treatment causes significant DNA degradation due to depyrimidination under the required acidic and thermal conditions [30].

To overcome these limitations, several bisulfite-free approaches have been developed. Among them, chemical-assisted pyridine borane sequencing (CAPS) is a promising strategy [31, 32]. Based on the selective oxidation of 5hmC to 5fC using a ruthenium compound and subsequent reduction with pyridine borane to dihydrouracil (DHU), 5hmC can be quantified directly. CAPS+ is an adapted version of CAPS involving oxidation of 5hmC to 5fC using 4-acetamido-2,2,6,6-tetramethylpiperidine-1-oxoammonium tetrafluoroborate (ACT^+^ BF_4_^−^), followed by Pinnick oxidation to convert 5fC to 5-carboxylcytosine (5caC), which is then reduced to DHU.

To the best of our knowledge, the performance of CAPS-derived and bisulfite-based approaches has not been compared in detail. We therefore aimed to assess accuracy, repeatability, lowest detectable and lowest quantifiable 5hmC levels, DNA loss, and DNA damage of established and adapted protocols. For bisulfite-based workflows, we tested two ruthenium-based oxidants and six thermal programs that differed in their total bisulfite incubation time. Among the bisulfite-free methods, we evaluated CAPS and CAPS+, the latter with two different reduction times. In addition, we developed a modified version of CAPS+, in which pic-borane reduction was replaced by bisulfite conversion (CAPS+&BS).

We targeted a 97 nt fragment of an *MGMT* enhancer (hs737) [33]. For method evaluation, we used three variants of a synthetic 97-mer oligonucleotide containing two CpG sites: variant “C” with unmodified cytosines; variant “mC” with methylated cytosines, and variant “hmC” with hydroxymethylated cytosines. In addition, genomic DNA from human hippocampal tissue was analyzed. Oligonucleotides and extracted genomic DNA were subjected to the respective treatment protocols and subsequently analyzed by PCR and pyrosequencing (PSQ).

## Methods

### Oligonucleotides and samples

**Fig. 1:**
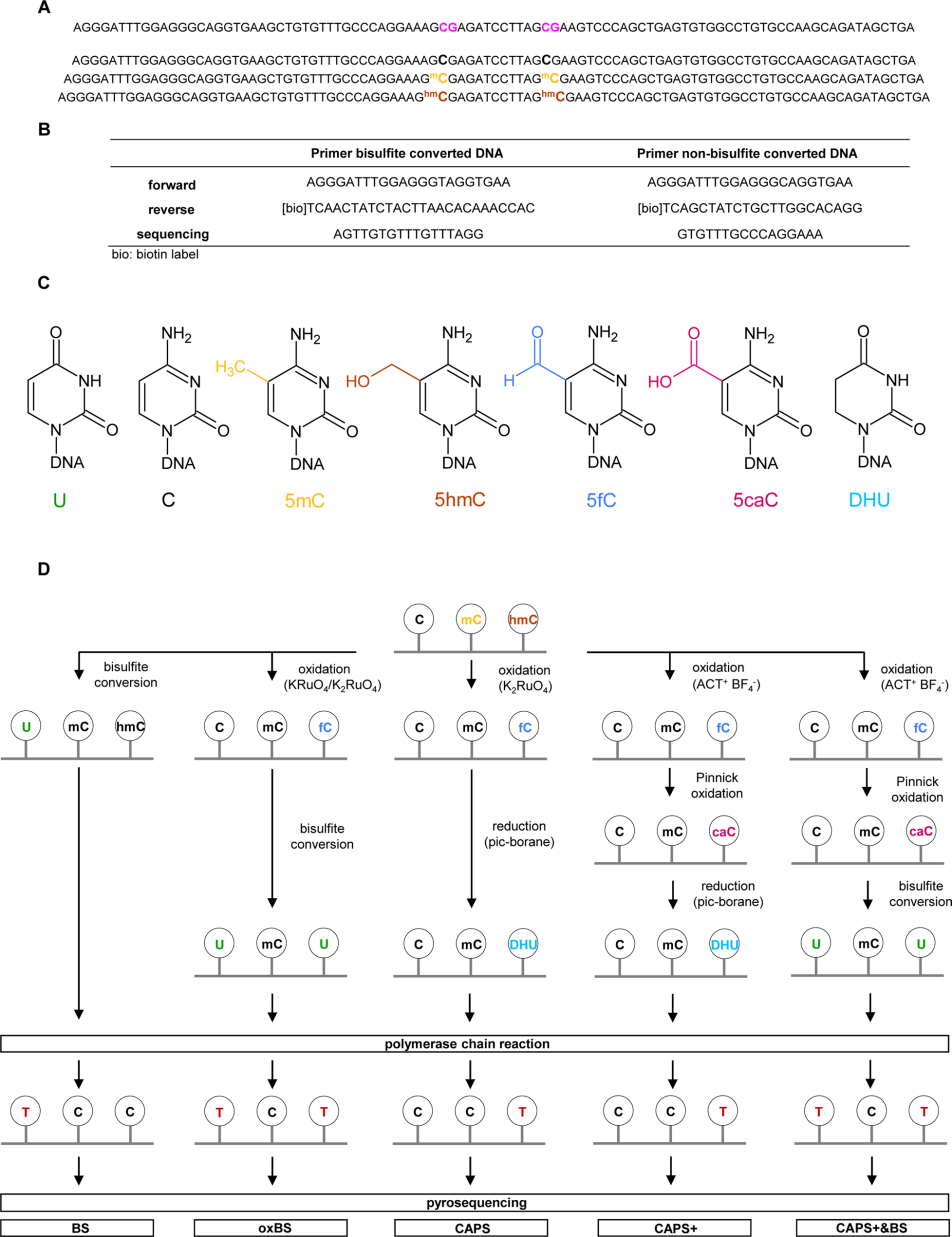
**A** Target sequence: a 97 nt fragment of an *MGMT* enhancer ((hs737) GRCh38.p14 chr10 NC_000010.11:128568604–128569741) containing two CpG sites. Three oligonucleotide variants (“C”, “mC”, and “hmC”) were used, carrying either cytosine, 5-methylcytosine, or 5-hydroxymethylcytosine at both CpG sites. **B** Primer sequences designed to amplify bisulfite-converted (BS, oxBS) or genomic (non-bisulfite treated) DNA. **C** Cytosine modifications. **D** Schematic overview of the applied treatment workflows. U: uracil, 5fC: 5-formylcytosine, 5caC: 5-carboxylcytosine, DHU: dihydroxyuracil, mC: 5-methylcytosine, hmC: 5-hydroxymethylcytosine. ACT^+^ BF4^-^: 4-acetamido-2,2,6,6-tetramethylpiperidine-1-oxoammonium tetrafluoroborate

A 97 nt fragment of an intergenic *MGMT* enhancer region containing two CpG sites (CpG 20 and CpG 21 of the enhancer) was selected as the target sequence. The enhancer sequence (hs737, GRCh38.p14 chr10 NC_000010.11:128568604–128569741) was obtained from the VISTA Enhancer Browser [34]. Synthetic 97-mer DNA oligonucleotides were ordered from Sigma Aldrich, the Stanford PAN Facility, and Eurogentec. Three variants were provided: variant “C”, containing unmodified cytosines at both CpG sites; variant “mC”, in which both CpG cytosines were methylated; and variant “hmC”, with hydroxymethylated cytosine at the two CpG sites (Fig. 1A). Lyophilized oligonucleotides were dissolved in nuclease-free water and serially diluted (1:150–1:1,500,000) from an initial DNA concentration of 5 ng/µL. Synthetic oligonucleotides served as standards and internal controls for method evaluation.

The applicability of the methods was further assessed using DNA extracted from human hippocampus tissue. Human hippocampal tissue samples were taken from human body donors enrolled in the body donor program of the Division of Anatomy of the Medical University of Vienna using standard anatomical approaches. All donors had voluntarily signed informed written consent prior to death. DNA was extracted using the DNeasy PowerMax Soil Kit (Qiagen). This DNA extraction kit was used since it allowed a higher sample input compared to DNA extraction kits commonly applied to human biological samples. DNA extraction was carried out according to the manufacturer’s instructions using 2.0 g of hippocampal sample. The eluted DNA was then concentrated by standard sodium acetate/ethanol precipitation as described by De Borre et al. [29]. In brief, 0.1 volumes of 3 M sodium acetate were mixed with the eluate, 2.5 volumes of ethanol were added, and the mixture was incubated at -20 °C for 60 min. After centrifugation at maximum speed (centrifuge 5424R, Eppendorf) for 20 min and removing the supernatant, the pellet was washed with cold 70% ethanol. After a further centrifugation step of 5 min, the supernatant was removed and the pellet air-dried and ultimately resuspended in 40 µL of ultra-pure water (ELGA).

### Bisulfite- and bisulfite free analysis of 5hmC

Bisulfite and oxidative bisulfite conversions were performed as described by De Borre et al. [29]. The CAPS protocol – including purification of genomic DNA, denaturation, oxidation, and pic-borane reduction – was adapted from Liu et al. [32]. CAPS+ was carried out according to Xu et al. [35]. The “CAPS+&BS” approach followed the CAPS+ protocol, except that pic-borane reduction was replaced by bisulfite conversion.

### Purification of DNA

Prior to any oxidation reaction, genomic DNA extracted from human hippocampus was purified using Bio-Rad Micro Bio-Spin P-6 SSC columns, following the manufacturer’s protocol but replacing SSC buffer with ELGA water. DNA cleanup after oxidation with either potassium perruthenate (KRuO_4_) or potassium ruthenate (K_2_RuO_4_) was performed using the same columns. For oligonucleotides, 3 µg of Carrier RNA (Qiagen) was added during cleanup to improve recovery. DNA purification following ACT^+^ BF4^-^ oxidation, Pinnick oxidation, or pic-borane reduction was carried out using Zymo-IC columns (Zymo Research) with Oligo Binding Buffer.

### DNA denaturation

Prior to oxidation reactions using KRuO_4_ or K_2_RuO_4_, DNA was denatured by adding 1.25 µL of 1 M NaOH to 21.75 µL of sample DNA and incubating at 37 °C for 30 min (ThermoMixer, Eppendorf). Amounts of 100 or 200 ng of purified human hippocampal DNA were used per reaction.

### Oxidation/reduction reactions

A 150 mM KRuO_4_ solution in 0.5 M NaOH was prepared as a 10x stock. The dark-brown solution was stored in single-use aliquots at -20 °C. Upon thawing, it appeared orange. After DNA denaturation, reactions were kept on ice for 3 min. For KRuO_4_ oxidation, 2 µL of the 10x solution were added, mixed, and incubated at 40 °C for 10 min, maintaining the orange color throughout the reaction.

K_2_RuO_4_ was generated by incubating the KRuO_4_ solution (150 mM) at 25 °C for two days with vortexing twice daily, as described by Zeng et al. [36]. The resulting deep red solution was stored in single-use aliquots at -20 °C. Upon thawing, it appeared orange. The 10x oxidant was diluted to 1x with ELGA water. 2.88 µL of the 1x oxidant were added to denatured DNA, followed by incubation at 37 °C and 850 rpm for one hour (ThermoMixer, Eppendorf). This step was repeated with an additional 2.88 µL of 1x oxidant under identical conditions.

ACT^+^ BF4^-^ was used to oxidize 5hmC to 5fC. DNA was incubated with 0.05 M sodium phosphate buffer (pH 7.5) and 0.05 M ACT^+^ BF4^-^ at 37 °C for four hours.

Pinnick oxidation was used to convert 5fC to 5caC. After ACT^+^ BF4^-^ oxidation, the reaction mix was further incubated with 0.2 M sodium acetate buffer (pH 4.3), 0.16 M NaClO_2_, and 1 M 2-methyl-2-butene at 25 °C for 16 h.

Pic-borane reduction of 5fC or 5caC into DHU was performed in a reaction containing 0.6 M 4-morpholineethanesulfonic acid (MES) and 0.2 M pic-borane at 37 °C and 850 rpm for two hours (ThermoMixer, Eppendorf).

### Bisulfite conversion

Bisulfite conversion was performed using the EpiTect Fast Bisulfite Conversion Kit (Qiagen), either directly or following oxidation. The bisulfite reaction mixture contained 40 µL of (oxidized) DNA solution, adjusted with nuclease-free water if necessary, 85 µL bisulfite solution and 15 µL DNA Protect Buffer. Thermal cycling programs were adapted from the manufacturer´s protocol (EpiTect Fast Bisulfite Conversion Handbook (Qiagen)), which includes two denaturation-incubation cycles totaling 30 min. In the adapted protocols, both the number of cycles and the incubation times were extended, with the longest program lasting 10 h. Each incubation step was preceded by a 5-minute denaturation at 95 °C. A summary of thermal programs (TPs) used is provided in Table 1. Bisulfite-treated DNA was cleaned up according to the manufacturer’s instructions, using Carrier RNA for oligonucleotides. DNA concentration of genomic samples was measured using a Qubit 4 fluorometer and the Qubit ssDNA Assay Kit (Thermo Scientific).

**Table 1:** Conditions of bisulfite conversion thermal programs (TP1–TP6)

Thermal program	No. of incubations	Incubation time [min]	Total incubation time [min]	Total program time [min]^1^
TP1	2	10	20	30
TP2	4	10	40	60
TP3	4	25, 45, 25, 45	140	160
TP4	6	25, 45, 75, 25, 45, 75	290	320
TP5	6	25, 65, 125, 25, 65, 125	430	460
TP6	6	25, 85, 175, 25, 85, 175	570	600

The table lists the number of incubations, duration of individual cycles, total incubation time, and total program time. In all programs, the incubation temperature was 60 °C, and each incubation step was preceded by a 5 min denaturation at 95 °C

^1^…including denaturation steps

### DNA amplification and pyrosequencing

Primer sets for non-bisulfite converted and bisulfite converted DNA were designed using PyroMark Assay Design Software 2.0.1.15 (Qiagen). Primer sequences are given in Fig. 1B. PCR amplification was performed at annealing temperatures of 61.2 °C and 60.7 °C, respectively, and an elongation temperature of 68.0 °C.

Human DNA methylation standards (Zymo Research) and mixtures thereof were used for the development and optimization of the assay enabling the analysis of bisulfite-treated DNA. For the development and optimization of the assay applied after bisulfite-free treatments, oligonucleotides containing C or T at the target CpG sites (Sigma Aldrich) and mixtures thereof were used.

PCR was run either on RotorGeneQ (Qiagen) or QuantStudio 5 (ThermoScientific) thermal cycler. No-template controls were included in all PCR runs to exclude contamination.

PCR products were then subjected to PSQanalysis using the PyroMarkQ48 Autoprep system, along with the PyroMarkQ48 Autoprep software 2.4.2, PyroMarkQ48 Autoprep Accessories, and PyroMarkQ48 Advanced CpG Reagents.

All treatment reactions were performed in triplicate unless stated otherwise. Due to limited availability of sample material, DNA from hippocampus tissue could not be subjected to all treatment reactions. Each replicate of treated DNA was analysed by PCR and PSQ.

### Method validation

Due to the low concentrations of the oligonucleotide solutions, fluorometric DNA quantification thereof was not feasible. Thus, recovery after bisulfite-based treatments was evaluated based on concentrations of genomic hippocampal DNA determined using Qubit ssDNA Assay Kit (Thermo Scientific). Due to measurement interferences, quantification after CAPS and CAPS + did not yield reliable data. Thus, recoveries of bisulfite-based and bisulfite-free methods were compared based on Ct-values obtained by PCR amplification (RotorGeneQ Series Software 2.3.1).

Lowest detectable levels (LDLs) and lowest quantifiable levels (LQLs) of 5hmC were assessed using the oligonucleotide containing unmethylated C by calculating mean and standard deviation (SD) of C/(C + T) ratios (bisulfite-based methods) or T/(C + T) ratios (bisulfite-free methods) of both CpG sites and adding three (LDL) or ten (LQL) times SD to the mean value. The theoretically expected value was 0% C (bisulfite-based treatments) or 0% T (bisulfite-free treatments).

### Data analysis

Data analysis and generation of graphics were performed using R software (version 4.4.2). To test for significant differences between two treatment conditions, Welch’s t-test followed by Benjamini-Hochberg (BH) p-value adjustment for multiple testing was applied. For testing between more than two groups, one-way ANOVA and post-hoc multiple comparisons were carried out using pairwise t-tests with BH p-value correction. p-values < 0.05 were considered to be statistically significant.

## Results

In this study, we performed a comparative validation of bisulfite-based and bisulfite-free treatment protocols for 5hmC analysis at single-CpG resolution.PSQ was chosen as a cost- and time-efficient platform for targeted CpG analysis.

In genomic regions with low CpG density, particularly in enhancers, 5hmC is relatively abundant [37]. Therefore, a 97 nt fragment of an *MGMT* enhancer (hs737) [33] containing two CpG sites was selected as the target region, providing a low-CpG-density context suitable for evaluating method performance under conditions relevant for enhancer-associated 5hmC. The performance of the treatment protocols was evaluated using three variants of a synthetic 97 nt oligonucleotide: variant “C”, “mC”, and “hmC”. To assess the applicability of the protocols to genomic DNA, DNA from human hippocampal tissue was analyzed, as brain tissue exhibits comparatively high 5hmC levels relative to other organs [38].

### Cytosine, 5-methylcytosine, and 5-hydroxymethylcytosine levels obtained for synthetic oligonucleotide variants

#### Bisulfite-based protocols

For bisulfite-based protocols, oligonucleotides were subjected to both conventional bisulfite conversion and oxidative bisulfite conversion. The additional oxidation step is required because bisulfite treatment of 5hmC leads to the formation of a stable cytosine-5-methylsulfonate (CMS) adduct that, similar to 5mC, is resistant to deamination [39]. Oxidation, typically carried out using KRuO_4_, converts 5hmC to 5-formylcytosine (5fC), which can subsequently be deformylated and deaminated to uracil during bisulfite treatment [28].

Compared to conventional bisulfite conversion of unmethylated cytosines, substantially longer incubation times are required to achieve efficient conversion of 5fC to uracil [40]. Total bisulfite incubation times of up to 570 min have been reported to ensure complete conversion of 5fC [40]. As even conventional bisulfite treatment causes considerable DNA degradation [30] and degradation increases with incubation time, shortening the incubation step is desirable [41].

We therefore evaluated five shorter thermal programs (TP1–TP5, Table 1) with total incubation times ranging from 20 to 430 min, in addition to a sixth program with a total incubation time of 570 min (TP6, Table 1) as proposed by Booth et al. [40]. For oxidative bisulfite conversion, two ruthenium-based oxidants were compared, KRuO_4_ and K_2_RuO_4_. K_2_RuO_4_ has been reported to be a milder and more efficient oxidant [36].

**Fig. 2:**
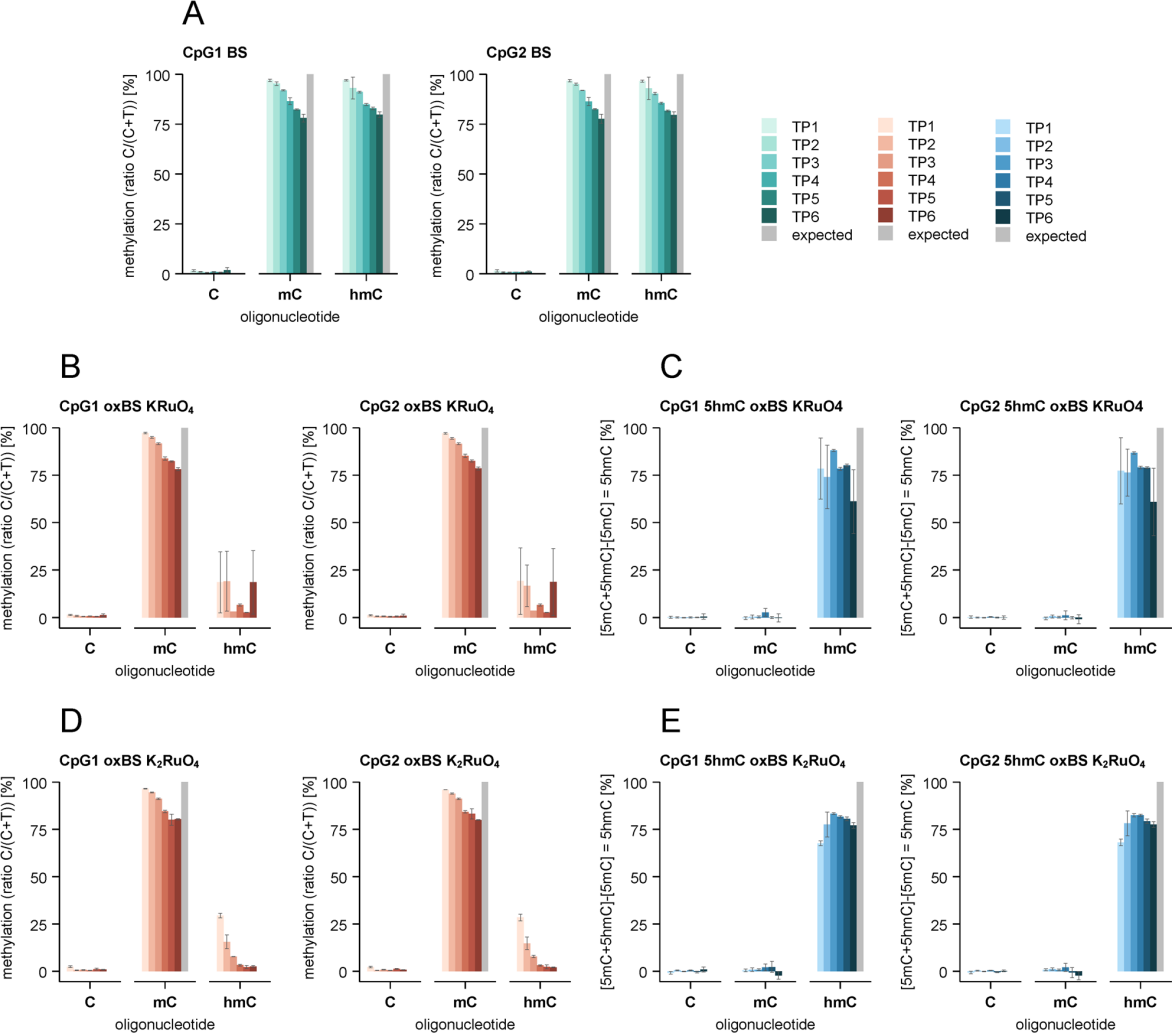
Determined C/(C + T) ratios and calculated 5hmC levels of oligonucleotides containing C, 5mC, or 5hmC at two distinct CpG sites for (**A**) BS, (**B**,** C**) oxBS using the oxidant KRuO_4_, and (**D**,** E**) oxBS using the oxidant K_2_RuO_4_ applying six different bisulfite conversion thermal programs (TP1–P6). Mean and standard deviation were calculated from three replicates. 5hmC levels were calculated subtracting mean values obtained for oxBS from mean values obtained for BS. The height of the grey bar indicates the expected C/(C + T) ratio. Turquoise: BS, red: oxBS, blue: calculated 5hmC levels

*Conventional bisulfite conversion*. Following bisulfite treatment and PCR amplification, unmethylated cytosines at both CpG sites are expected to be sequenced as thymine, whereas 5mC and 5hmC should be retained as cytosine. Across all six thermal programs, unmethylated cytosines were almost completely converted to uracil, as indicated by the consistently low C/(C + T) ratios obtained for the “C” oligonucleotide (Fig. 2A). In contrast, preservation of 5mC and 5hmC strongly depended on bisulfite incubation time, with increasing incubation times yielding lower C/(C + T) ratios (Fig. 2A).

For the “mC” oligonucleotide, TP1 yielded significantly higher C/(C + T) ratios (97.0% ± 0.6% and 96.7% ± 0.6%) compared with TP2 (95.3% ± 0.9% and 95.0% ± 0.6%, *p* < 0.05), TP3 (91.9% ± 0.3% and 91.9% ± 0.1%, *p* < 0.001), TP4 (86.5% ± 1.7% and 86.4% ± 2.1%, *p* < 0.05), TP5 (82.3% ± 0.3% and 82.5% ± 0.3%, *p* < 0.001) and TP6 (78.0% ± 2.0% and 77.7% ± 2.3%, *p* < 0.001) at CpG 1 and CpG 2, respectively. A similar pattern was observed for the “hmC” variant. TP1 resulted in significantly higher C/(C + T) ratios (97.1% ± 0.4% and 96.6% ± 0.6%) compared with TP3 (91.1% ± 0.4% and 90.3% ± 0.6%, *p* < 0.001), TP4 (84.9% ± 0.4% and 85.8% ± 0.4%, *p* < 0.001), TP5 (82.9% ± 0.7% and 81.6% ± 0.5%, *p* < 0.001), and TP6 (79.7% ± 1.5% and 79.6% ± 1.5%, *p* < 0.001).

*Oxidative bisulfite conversion.* After efficient oxidative bisulfite treatment and subsequent PCR, unmethylated cytosines and 5hmC at the two CpG sites should be sequenced as T, whereas 5mC should remain unaffected and be sequenced as C. The low C/(C + T) ratios obtained for the oligonucleotide containing unmethylated C indicate that conversion to uracil was almost complete, independent of the thermal program (Figs. 2B, D) (TP1: 1.4% ± 0.3% and 1.3% ± 0.3%, TP2: 0.9% ± 0.3% and 0.7% ± 0.1%, TP3: 0.7% ± 0.1% and 0.8% ± 0.1%, TP4: 0.8% ± 0.1% and 0.5% ± 0.2%, TP5: 0.6% ± 0.1% and 0.7% ± 0.2%, TP6: 1.2% ± 0.7% and 1.0% ± 0.8%, at CpG 1 and CpG 2, respectively for oxidation using KRuO_4_ (Fig. 2B) and TP1: 2.4% ± 0.5% and 2.2% ± 0.4%, TP2: 0.5% ± 0.2% and 0.5% ± 0.1%, TP3: 0.7% ± 0.2% and 0.8% ± 0.1%, TP4: 0.5% ± 0.1% and 0.4% ± 0.03%, TP5: 1.2% ± 0.4% and 1.2% ± 0.2%, TP6: 0.9% ± 0.1% and 0.8% ± 0.1%, at CpG 1 and CpG 2, respectively, for oxidation using K_2_RuO_4_ (Fig. 2D)).

For 5mC, the same trend as in conventional bisulfite conversion was observed: C/(C + T) ratios decreased with increasing bisulfite incubation time (Figs. 2B, D). This trend held true for both oxidants, KRuO_4_ (Fig. 2B) and K_2_RuO_4_ (Fig. 2D). After oxidation with KRuO_4_, TP1 resulted in significantly higher proportions of C (97.2% ± 0.5% and 97.1% ± 0.4%) than TP2 (95.0% ± 0.5% and 94.5% ± 0.5%, *p* < 0.001), TP3 (91.6% ± 0.5% both, *p* < 0.001), TP4 (83.8% ± 0.9% and 85.2% ± 0.9%, *p* < 0.01), TP5 (82.3% ± 0.4% and 82.5% ± 0.5%, *p* < 0.001) and TP6 (78.1% ± 0.8% and 78.5% ± 0.8%, *p* < 0.001) at the two CpG sites, respectively. Following oxidation with K_2_RuO_4_, TP1 again resulted in significantly higher C/(C + T) ratios (96.5% ± 0.2% and 96.0% ± 0.1%) compared to TP2 (94.5% ± 0.3% and 93.9% ± 0.3%, *p* < 0.01), TP3 (91.1% ± 0.4% and 91.2% ± 0.4%, *p* < 0.01), TP4 (84.5% ± 0.6% and 84.3% ± 0.7%, *p* < 0.01), TP5 (80.1% ± 2.9% and 83.2% ± 2.7%, *p* < 0.05), and TP6 (80.3% ± 0.4% and 80.0% ± 0.1%, *p* < 0.001) at the two CpG sites.

For 5hmC, the type of oxidant had a strong impact. Oxidation with KRuO_4_ did not lead to repeatable C/(C + T) ratios (Fig. 2B). In contrast, oxidation with K_2_RuO_4_ showed a clear trend: the longer the bisulfite incubation, the lower were the C/(C + T) ratios, reflecting efficient oxidation of 5hmC to 5fC and subsequent conversion to uracil. TP1 yielded significantly higher C/(C + T) ratios (29.4% ± 1.2% and 28.4% ± 1.7%) than TP2 (15.6% ± 3.6% and 14.7% ± 3.4%, *p* < 0.05), TP3 (7.7% ± 0.1% and 7.8% ± 0.7%, *p* < 0.01), TP4 (3.2% ± 0.5% and 3.1% ± 0.3%, *p* < 0.01), TP5 (2.2% ± 0.8% and 2.3% ± 1.0%, *p* < 0.001), and TP6 (2.6% ± 0.4% and 2.1% ± 0.1%, *p* < 0.01) at the two CpG sites.

The resulting 5hmC levels, calculated by subtracting the mean values obtained by oxBS from those obtained by BS, are shown in Fig. 2C (KRuO_4_) and 2E (K_2_RuO_4_). Standard deviations were evaluated by calculating the combined uncertainty from the two individual experiments using the square root of the sum of squares method. Due to low repeatability, values obtained after oxidation with KRuO_4_ are expected to be relatively inaccurate. Using K_2_RuO_4_, the calculated 5hmC levels were more reliable, although they remained far below 100%. 5hmC levels were 67.6% ± 1.3% and 68.1% ± 1.8% (TP1), 77.5% ± 6.5% and 78.2% ± 6.6% (TP2), 83.3% ± 0.4% and 82.5% ± 0.9% (TP3), 81.6% ± 0.7% and 82.5% ± 0.5% (TP4), 80.6% ± 1.0% and 79.3% ± 1.1% (TP5) and 77.2% ± 1.5% and 77.6% ± 1.5% (TP6) at CpG1 and CpG2, respectively. 5hmC values increased from TP1 to TP3, and decreased thereafter.

When calculating the difference to obtain 5hmC levels, negative values were also received for oligonucleotides not containing 5hmC: -0.1% ± 2.1% and − 0.80% ± 2.4% for the “mC” variant at TP6 using KRuO_4_ (Fig. 2C) and − 2.3% ± 2.0% and − 2.3% ± 2.3% at the two CpG sites, respectively at TP6 using K_2_RuO_4_ (Fig. 2E).

### Bisulfite-free protocols

Unlike bisulfite-based approaches, the bisulfite-free workflows tested in this study allow direct quantification of 5hmC. CAPS involves selective oxidation of 5hmC to 5fC, which is then reduced to DHU by pyridine borane through deformylation/deamination [31, 32]. DHU is then amplified and sequenced as T. In their first study [31], Liu et al. used KRuO_4_ which was later replaced by K_2_RuO_4_ [32]. They also reported that -borane (pic-borane) yields higher hmC to T conversion rates than pyridine borane.

CAPS + is an adapted version of CAPS, in which 5hmC is oxidized to 5fC using 4-acetamido-2,2,6,6-tetramethylpiperidine-1-oxoammonium tetrafluoroborate (ACT^+^ BF4^−^, Bobbitt’s salt), followed by Pinnick oxidation to convert 5fC to 5-carboxylcytosine (5caC), which is then reduced to DHU by pic-borane. Unlike ruthenium-based oxidation, which requires denaturation of double-stranded DNA (dsDNA) to single-stranded DNA (ssDNA) [28], ACT^+^ BF4^−^ can be applied directly to dsDNA [35].

**Fig. 3:**
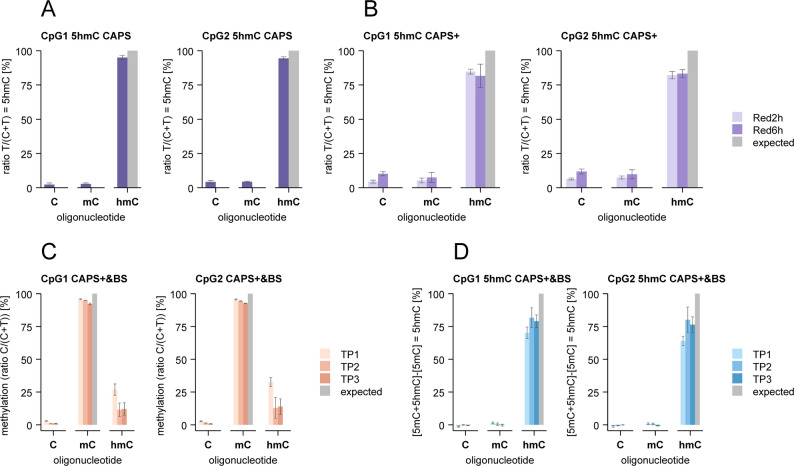
**A**,** B** Determined T/(C + T) ratios of oligonucleotides containing C, 5mC, or 5hmC at two distinct CpG sites undergoing (**A**) CAPS and (**B**) CAPS+ (applying a 2- or 6-hour reduction time). Determined C/(C + T) ratios (**C**) and calculated 5hmC levels (**D**) of oligonucleotides undergoing CAPS+&BS applying thermal programs TP1–TP3. Mean and standard deviation were calculated from three replicates. 5hmC levels were calculated subtracting mean values obtained for CAPS+&BS from mean values obtained for BS. The height of the grey bar indicates the expected T/(C + T) or C/(C + T) ratio. Purple: CAPS/CAPS+, red: CAPS+&BS, blue: calculated 5hmC levels

With both CAPS and CAPS+, unmethylated cytosine and 5mC at the two CpG sites should be sequenced as C, whereas 5hmC should be sequenced as T. For the “hmC” variant, CAPS resulted in very efficient conversion, with T/(C + T) ratios of 95.0% ±1.6% and 94.4% ± 1.2% at the two CpG sites, respectively (Fig. 3A). However, some low T/(C + T) ratios, reflecting false positives, were also observed for oligonucleotides containing unmethylated cytosine or 5mC (Fig. 3A). In detail, we observed 2.3% ± 1.2% and 4.2% ± 1.1%, and 2.7% ± 0.7% and 4.3% ± 0.5% of sequenced T at the two CpG sites for the unmethylated or the “mC” oligonucleotide, respectively.

For CAPS+, we tested two pic-borane reduction times, 2 h and 6 h. The 2 h reduction yielded T/(C + T) ratios of 84.7% ± 1.8% and 82.2% ± 2.7% at CpG1 and CpG2. After 6 h, T/(C + T) ratios for the “hmC” variant (81.6% ± 8.6% and 83.2% ± 3.02%) did not differ significantly from the 2 h treatment (Fig. 3B). CAPS+ also yielded false positives with T/(C + T) ratios of 4.4% ± 1.1% and 6.4% ± 0.7%, 10.2% ± 1.5% and 11.8% ± 1.8% for the oligonucleotide containing unmethylated cytosines at the two CpG sites, for reduction times of 2 and 6 h, respectively. Here, false positive rate was significantly higher for the 6 h reduction compared to 2 h reduction (*p* < 0.004). For the oligonucleotide containing mC, obtained T/(C + T) ratios were 5.3% ± 1.8% and 7.5% ± 1.1%, and 7.5% ± 3.7% and 9.8% ± 3.3% at the two CpG sites, for reduction times of 2 and 6 h, respectively. Overall, 5hmC levels determined by CAPS+ were significantly lower than those obtained with CAPS (*p* < 0.05).

### Combined approach “CAPS+&BS”

In addition to bisulfite-based and bisulfite-free protocols, we tested a combined approach. Xu et al. proposed that 5caC might serve as a better substrate for bisulfite conversion than 5fC, potentially allowing shorter bisulfite incubation times [35]. To our knowledge, this approach has not been tested experimentally. Our combined protocol, CAPS+&BS, was identical to CAPS+, except that pic-borane reduction was replaced by bisulfite conversion. Thermal programs TP1–TP3 were tested (Fig. 3C).

Unmethylated cytosine was almost fully converted to T, as indicated by low C/(C + T) ratios (3.0% ± 0.2% and 2.7% ± 0.3% (TP1), 1.1% ± 0.03% and 1.4% ± 0.1% (TP2), 1.1% ± 0.1% and 0.8% ± 0.1% (TP3) at the two CpG sites, respectively. For 5mC, C/(C + T) ratios decreased with longer bisulfite incubation, from 95.7% ± 0.4% and 95.6% ± 0.3% (TP1) to 94.9% ± 0.1% and 94.4% ± 0.2% (TP2, *p* < 0.05), to 92.2% ± 0.7% and 92.6% ± 0.1% (TP3, *p* ≤ 0.01) at the two CpG sites.

For 5hmC, C/(C + T) ratios were highest for TP1 (26.8% ± 4.3% and 32.6% ± 3.5%), and lower for TP2 (11.4% ± 5.2% and 12.9% ± 7.9%, *p* < 0.05) and TP3 (12.0% ± 4.7% and 13.9% ± 5.8%, *p* < 0.05). The resulting 5hmC levels calculated by subtraction were 70.2% ± 4.3% and 64.0% ± 3.5% (TP1), 81.7% ± 7.2% and 80.1% ± 9.8% (TP2), and 79.1% ± 4.7% and 76.4% ± 5.9% (TP3) at the two CpG sites, respectively (Fig. 3D). Negative values were obtained when forming the difference for the unmethylated cytosine: -1.4% ± 0.5% and − 1.2% ± 0.7% (TP1), -0.1% ± 0.2% and − 0.6% ± 0.2% (TP2), -0.4% ± 0.2% and − 0.1% ± 0.1% (TP3), and the “mC” variant: -0.2% ± 0.7% and − 0.8% ± 0.2% (TP3) at the two CpG sites, respectively.

### Lowest detectable and lowest quantifiable 5hmC levels

We assessed the lowest detectable (LDL) and lowest quantifiable (LQL) 5hmC levels by analyzing the oligonucleotide variant “C” in triplicates. LDL and LQL were calculated as the mean + 3x SD or 10x SD of the C/(C + T) ratios (bisulfite-based protocols) or T/(C + T) ratios (bisulfite-free protocols) at both CpG sites. Ratios of 0% indicate complete conversion, whereas higher values reflect false positives.

Overall, bisulfite-free protocols (CAPS and CAPS+) showed higher LDL and LQL values compared to bisulfite-based protocols and the combined approach (Table 2). Among bisulfite-based protocols, the lowest LDL and LQL values were obtained with thermal program TP3 (total incubation time 140 min). The type of oxidant (KRuO_4_ or K_2_RuO_4_) had no significant impact on LDL and LQL values. Similar LDL and LQL values were obtained for the combined CAPS+&BS approach using TP3.

For bisulfite-free protocols, CAPS and CAPS+ with 2 h reduction yielded comparable LDL and LQL values, whereas CAPS+ with 6 h reduction showed the highest LDL and LQL values of all protocols tested.

**Table 2:** Lowest detectable (LDL) and lowest quantifiable (LQL) 5hmC levels (%) for the different treatment approaches

Method									
oxBS KRuO_4_									
	**TP1**	**TP2**		**TP3**		**TP4**	**TP5**		**TP6**
LDL (%)	1.95	1.02		0.43		1.04	0.63		4.06
LQL (%)	5.97	3.20		1.55		3.00	1.91		12.83

oxBS K_2_RuO_4_									
	**TP1**	**TP2**		**TP3**		**TP4**	**TP5**		**TP6**
LDL (%)	1.29	1.14		0.45		0.98	0.56		3.66
LQL (%)	6.02	2.89		1.71		2.27	2.90		10.84

CAPS									
LDL (%)	7.63								
LQL (%)	17.82								

CAPS+									
	Reduction 2 h				Reduction 6 h				
LDL (%)	9.36				16.42				
LQL (%)	18.62				29.04				

CAPS+&BS									
			**TP1**		**TP2**			**TP3**	
LDL (%)			0.44		0.43			0.34	
LQL (%)			4.47		2.25			1.75	

### Cytosine, 5-methylcytosine, and 5-hydroxymethylcytosine levels for genomic DNA samples

To evaluate applicability to genomic DNA, selected bisulfite-based, bisulfite-free, and combined protocols were applied to human hippocampal tissue. Theoretical 5hmC levels at the target CpG sites in this tissue were, however, not available.

### Bisulfite-based protocols

**Fig. 4:**
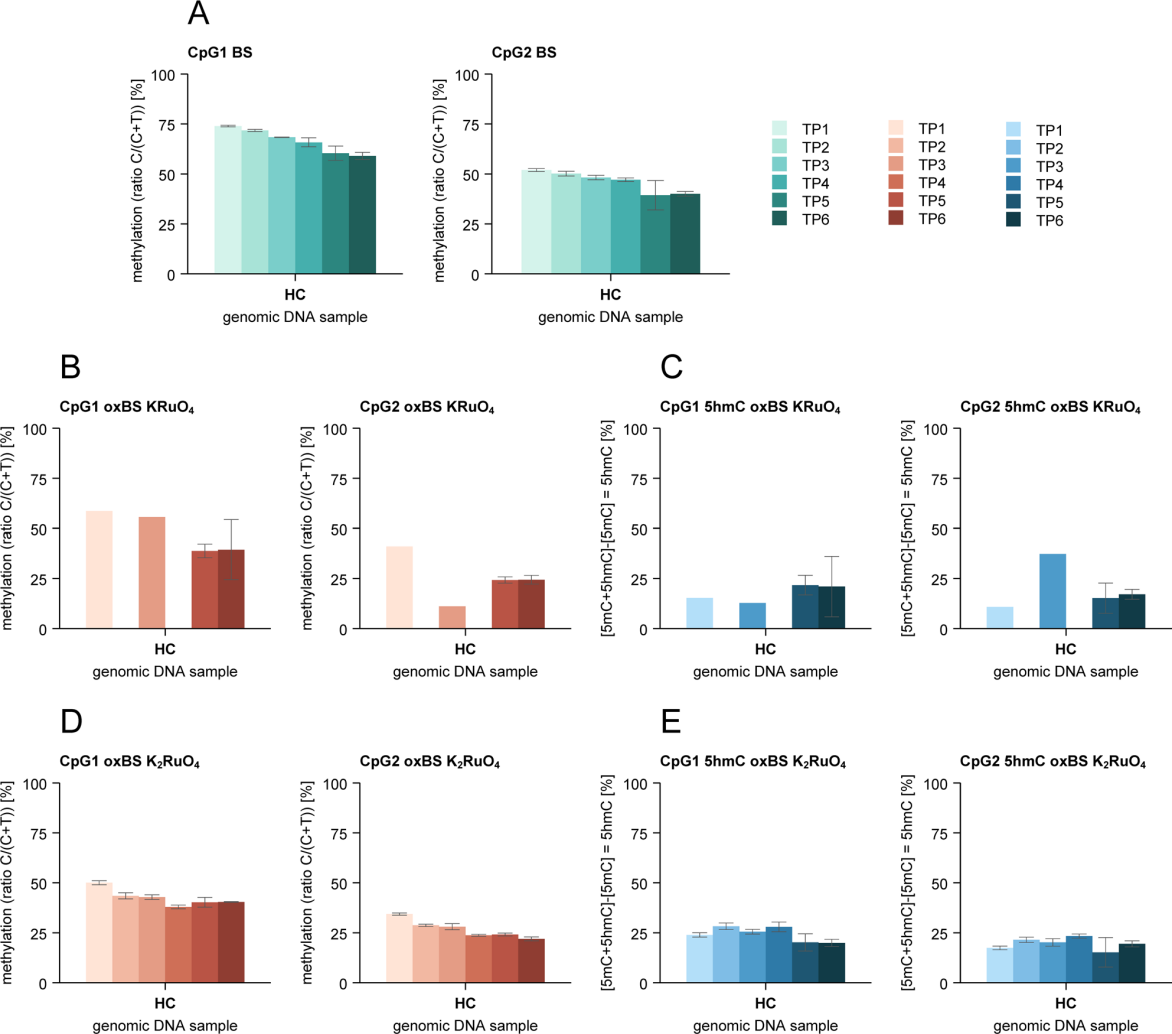
Determined C/(C + T) ratio and calculated 5hmC levels of genomic DNA from hippocampus (HC) for (**A**) BS, (**B**,** C**) oxBS using the oxidant KRuO_4_, and (**D**,** E**) oxBS using the oxidant K_2_RuO_4_, applying six different bisulfite conversion thermal programs (TP1–P6). Mean of at least two replicates. Standard deviation given if at least three replicates were available. 5hmC levels were calculated by subtracting mean values obtained for oxBS from mean values obtained for BS. Turquoise: BS, red: oxBS, blue: calculated 5hmC levels

For conventional bisulfite conversion, the same trend as for the oligonucleotide variants “C” and “mC” was observed: C/(C + T) ratios decreased with longer bisulfite incubation. TP6 yielded significantly lower C/(C + T) ratios than TP1–TP5 (*p* ≤ 0.020) (Fig. 4A).

For oxBS, C/(C + T) ratios obtained with KRuO_4_ were less repeatable than those obtained with K_2_RuO_4_. Using K_2_RuO_4_, significantly lower C/(C + T) ratios were obtained with TP2–TP5 compared to TP1 (*p* ≤ 0.012) (Figs. 4B, D). 5mC levels, obtained directly from oxBS, were 50.0% ± 1.0% and 34.3% ± 0.6% (TP1), 43.5% ± 1.5% and 28.7% ± 0.6% (TP2), 42.8% ± 1.2% and 28.0% ± 1.5% (TP3), 37.9% ± 0.9% and 23.8% ± 0.5% (TP4), 40.2% ± 2.4% and 24.1% ± 0.7% (TP5), 40.4% ± 0.2% and 22.0% ± 1.0% (TP6) at the two CpG sites, respectively.

Calculated 5hmC levels were 23.9% ± 1.1% and 17.4% ± 0.9% (TP1), 28.3% ± 1.6% and 21.5% ± 1.3% (TP2), 25.6% ± 1.2% and 20.2% ± 1.9% (TP3), 28.0% ± 2.4% and 23.4% ± 1.0% (TP4), 20.2% ± 4.3% and 15.2% ± 7.4% (TP5) and 20.0% ± 1.8% and 19.5% ±1.5% (TP6) at the two CpG sites, respectively.

### Bisulfite-free protocols

**Fig. 5:**
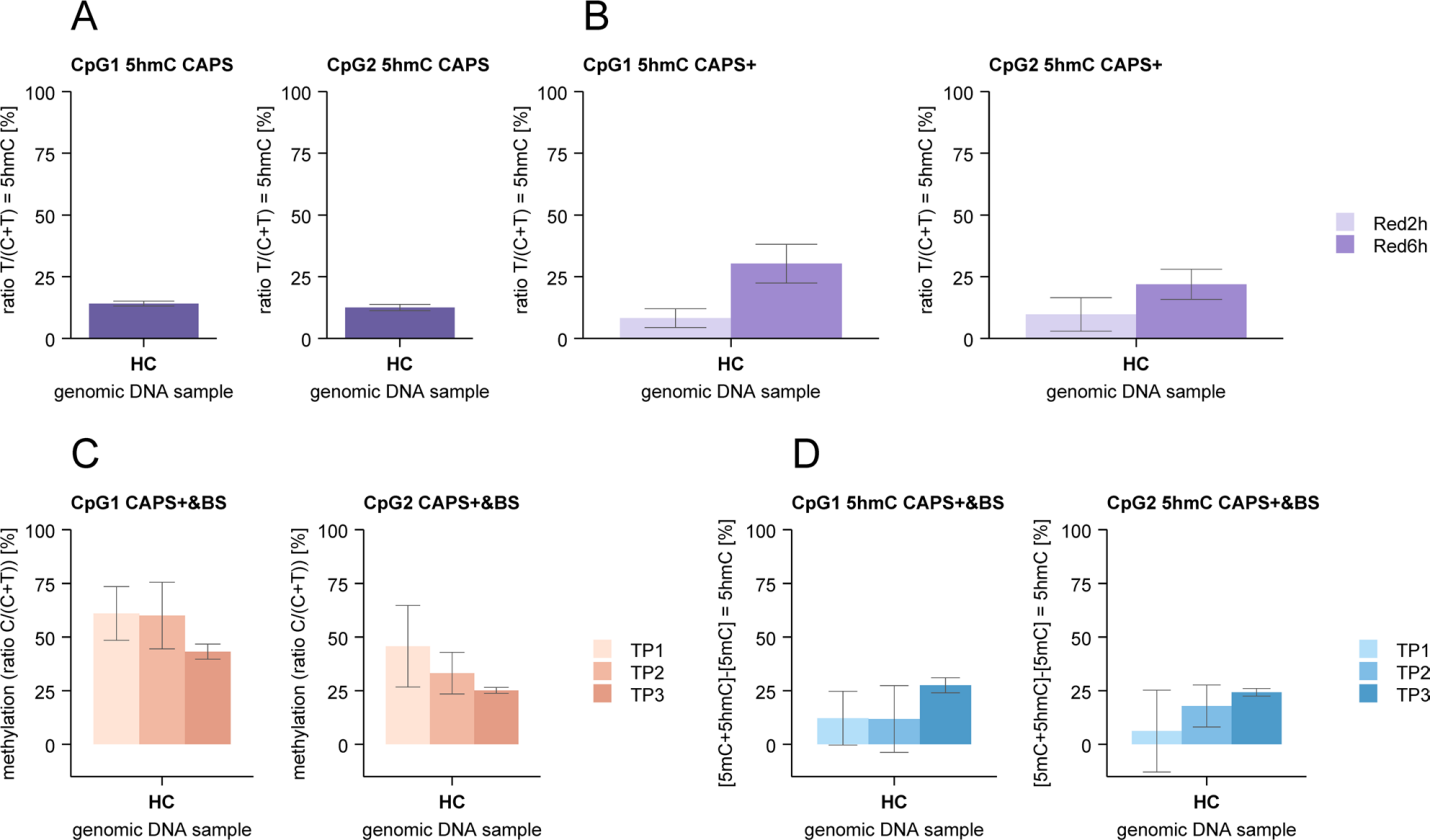
**A**,** B** Determined T/(C + T) ratios of hippocampal DNA sample (HC) undergoing (**A**) CAPS and (**B**) CAPS+ (applying a 2 or 6 h reduction time). Determined C/(C + T) ratios (**C**) and calculated 5hmC levels (**D**) of HC undergoing CAPS+&BS applying thermal programs TP1–TP3. Mean and standard deviation were calculated from three replicates. 5hmC levels were calculated by subtracting mean values obtained for CAPS+&BS from mean values obtained for BS. Purple: CAPS/CAPS+, red: CAPS+&BS, blue: calculated 5hmC levels

After CAPS, 5hmC levels at both CpG sites were above the LDL (7.63%) but below the LQL (17.82%) (Fig. 5A). With CAPS + and 2 h reduction, 5hmC level of the first CpG was < 9.36%, that of the second CpG between LDL (9.36%) and LQL (18.62%). CAPS+ with 6 h reduction yielded 5hmC level of 30.3 ± 7.9% for the first CpG, whereas that of the second CpG was between LDL and LQL (Fig. 5B).

### Combined approach “CAPS+&BS”

For the combined approach CAPS+&BS, C/(C + T) ratios decreased with longer bisulfite incubation, similar to the bisulfite-based protocols. However, repetability was relatively low, and differences between TP1–TP3 were not significant (Fig. 5C). Accordingly, calculated 5hmC levels showed high standard deviations and were as follows: 12.2% ± 12.5% and 6.2% ± 19.0% (TP1), 11.8% ± 15.6% and 17.9% ± 9.8% (TP2), and 27.5% ± 3.5% and 24.2% ± 1.8% (TP3).

### DNA recovery

Harsh conditions during bisulfite conversion lead to DNA degradation, but even milder bisulfite-free protocols can reduce DNA recovery, for example due to loss of DNA during purification steps.

For conventional bisulfite conversion, we assessed the impact of incubation time on DNA recovery, while for oxidative bisulfite conversion, we also considered the effect of oxidant type. DNA recovery was assessed by determining hippocampal DNA concentration before and after treatment. This could not be performed on oligonucleotide solutions, as their concentrations were below the LOD of the Qubit instrument.

**Fig. 6:**
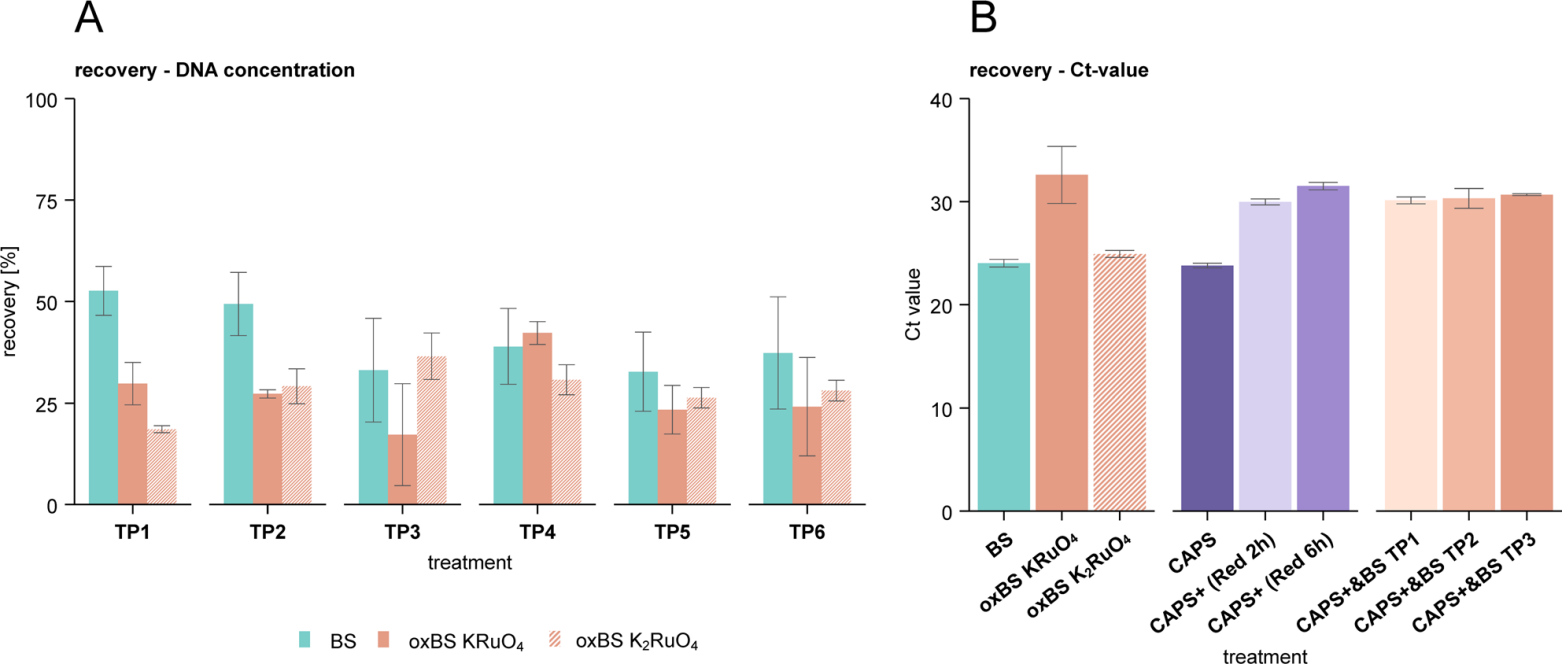
Recovery of DNA after the respective treatments based on (**A**) measured ssDNA concentration of genomic samples and (**B**) Ct-values. Ct-values for BS and oxBS (both KRuO_4_ and K_2_RuO_4_) refer to TP3. TP: thermal program, Red: reduction. Mean and standard deviation were calculated from three replicates

In general, the oxidation step preceding bisulfite conversion reduced DNA recovery. For TP1 (*p* < 0.001) and TP2 (*p* < 0.01), recovery was significantly lower when oxidation preceded bisulfite treatment (Fig. 6A), this hold true for both KRuO_4_ and K_2_RuO_4_. For TP1, DNA recovery with K_2_RuO_4_ was significantly lower than that with KRuO_4_ (*p* < 0.05). For CAPS+&BS, DNA loss was so substantial that DNA concentration fell below LOD of the Qubit instrument. Ct-values from PCR amplification were therefore used to compare DNA recovery across protocols (Fig. 6B). Notably, low DNA concentrations determined fluorometrically reflect DNA loss during treatment, whereas high Ct-values obtained in PCR indicate DNA loss and/or DNA damage.

Among all protocols, lowest Ct-values were observed for conventional bisulfite conversion and CAPS using K_2_RuO_4_. Comparing the ruthenium-based oxidants, K_2_RuO_4_ outperformed KRuO_4_ due to lower DNA damage, with Ct-values of 24.9 ± 0.3 (oxBS with K_2_RuO_4_, TP3) vs. 32.6 ± 2.8 (oxBS with KRuO_4_, TP3; *p* < 0.001).

CAPS+ led to higher Ct-values than CAPS, irrespective of pic-borane reduction time (2 or 6 h, *p* < 0.001). TP1–TP3 of CAPS+&BS yielded significantly higher Ct-values (*p* < 0.001) than BS, oxBS with K_2_RuO_4_ (TP3), and CAPS.

## Discussion

We compared bisulfite-based and bisulfite-free treatment protocols for 5hmC analysis at single-CpG resolution with respect to accuracy, repeatability, lowest detectable and lowest quantifiable 5hmC levels, and DNA recovery. Both approaches have previously been applied in combination with various sequencing platforms, including DNA methylation arrays [29, 42]. We selected PSQas a cost- and time-efficient technique for targeted sequencing applications, such as DNA methylation analysis at specific CpG sites [43]. Comparative validation was performed on a 97 nt fragment of an *MGMT* enhancer (hs737) [33] containing two CpG sites, using synthetic oligonucleotides and genomic DNA from human hippocampal tissue.

With 5hmC levels of approximately 95% obtained for the “hmC” variant of the 97-mer oligonucleotide, CAPS, which involves selective oxidation of 5hmC to 5fC followed by reduction of 5fC to DHU, outperformed all other treatment protocols with regard to accuracy and repetability. CAPS+, in which 5hmC is oxidized to 5caC via 5fC prior to reduction to DHU, as well as all bisulfite-based protocols, resulted in significantly lower 5hmC levels for the “hmC” oligonucleotide. For bisulfite-based approaches, total bisulfite incubation time had a pronounced effect on accuracy. The highest accuracy was achieved with a total incubation time of 140 min, which is substantially shorter than the 570 min proposed in literature [40]. Notably, in our experiments, increasing incubation times affected C/(C + T) ratios in conventional bisulfite conversion and oxidative bisulfite conversion to a similar extent. This observation suggests that under prolonged bisulfite incubation conditions, not only cytosine but also 5mC, 5hmC, and 5fC may be converted to uracil, leading to false negative 5hmC results.

Among the two ruthenium-based oxidants evaluated for oxBS, K_2_RuO_4_ turned out to be more suitable than KRuO_4_, as it resulted in more repeatable 5hmC levels. K_2_RuO_4_ has previously been reported to be a milder and more efficient oxidant than KRuO_4_ [36]. In general, oxidation efficiency can be compromised by impurities in reagents. In our experiments, nuclease-free water was found to be insufficiently pure and had to be replaced by ultrapure water (ELGA). In addition, high purity of genomic DNA was critical for achieving high oxidation efficiency, in agreement with previous studies [40].

False positive signals were negligible for bisulfite-based protocols, as unmethylated cytosines were almost completely converted to uracil across all six thermal programs tested. Among the bisulfite-based protocols, the lowest LDL and LQL values were obtained with thermal program TP3, corresponding to a total incubation time of 140 min. Notably, the choice of oxidant (KRuO_4_ or K_2_RuO_4_) had no significant impact on LDL and LQL values.

Both CAPS and CAPS+ were associated with higher false-positive rates than bisulfite-based protocols. Although for CAPS and CAPS+, the “C” and “mC” oligonucleotide variants should theoretically yield T/(C + T) ratios of 0%, T/(C + T) ratios deviating from 0% were consistently observed. As a consequence of these false-positive ratios, CAPS and CAPS+ showed higher LDL and LQL values than bisulfite-based protocols. CAPS+ with a reduction time of 6 h yielded the highest LDL and LQL values among all protocols investigated. Although CAPS + has previously been reported to improve conversion efficiency and reduce false-positive rates compared to CAPS [35], this advantage was not reflected in our experimentally determined LDL and LQL values under the conditions tested here.

The observed false-positive T signals in CAPS and CAPS+ likely arise from chemical side reactions during oxidation and/or reduction steps. In particular, prolonged pic-borane reduction in CAPS+ significantly increased background signals, suggesting that extended exposure to reducing conditions may promote non-specific modification or partial conversion of unmodified cytosine or 5mC. Similarly, the relatively harsh oxidative conditions in ruthenium-based CAPS, which require prior denaturation of dsDNA, may increase chemical susceptibility of cytosines and contribute to low-level background signals. Although the exact molecular mechanisms underlying these false positives remain to be fully elucidated, our data indicate that reaction conditions critically influence background levels and thus directly affect LDL and LQL values.

A limitation of the present study is that the analytical validation of false-positive rates was primarily performed using ss oligonucleotides. Therefore, the extent to which these background signals translate to dsDNA, which more closely reflects the structure of genomic DNA, cannot be directly inferred. Differences between ss and dsDNA may arise from reduced accessibility of bases within the double helix, reannealing effects, or the formation of secondary structures, which can influence chemical reactivity and conversion efficiency. Such effects are expected to be more relevant for bisulfite-free approaches that rely on direct chemical or enzymatic modification of cytosine derivatives, whereas bisulfite-based protocols involve a denaturation step and largely operate on ssDNA during conversion. However, it is important to note that false-positive signals for CAPS and CAPS+ were already observed under these controlled conditions, whereas bisulfite-based protocols showed negligible background. This suggests that the observed differences in analytical performance are largely driven by intrinsic properties of the respective methods, as they are already evident under controlled ss conditions. In addition, the applicability of the protocols to genomic DNA was confirmed using hippocampal tissue samples.

High LDL and LQL values represent a major limitation for 5hmC analysis, due to the generally low abundance of this modification in most tissues. We therefore evaluated the applicability of the different treatment protocols to genomic DNA from human hippocampal tissue. Although brain tissue is known to exhibit comparatively high 5hmC levels, the determined values were below the LDL and/or LQL of CAPS and CAPS+. In contrast, bisulfite-based treatment using TP3 resulted in 5hmC levels of 25.6% and 20.2% at the two target CpG sites. These 5hmC levels appear plausible, however, no reference values were available for direct comparison.

DNA recovery across treatment protocols was assessed using Ct-values obtained from PCR amplification of treated hippocampal DNA. Among all protocols, the lowest Ct-values, indicating the highest DNA recovery, were observed after conventional bisulfite conversion and CAPS using K_2_RuO_4_. OxBS with K_2_RuO_4_ resulted in significantly lower Ct-values than oxBS with KRuO_4_, consistent with previous findings showing reduced DNA degradation for K_2_RuO_4_ [36]. Notably, CAPS+ led to higher Ct-values than CAPS, despite the fact that ACT^+^ BF4^-^ can be applied directly to dsDNA, while ruthenium-based oxidation requires prior denaturation. We hypothesize that the lower DNA recovery of CAPS+ is primarily caused by the higher number of treatment and purification steps involved.

DNA recovery of bisulfite-based protocols was also evaluated by determining hippocampal DNA concentration before and after treatment. Bisulfite conversion resulted in a significant reduction in DNA concentration. For the shortest thermal program, the oxidation step preceding bisulfite conversion significantly reduced DNA recovery, irrespective of the ruthenium-based oxidant used. Published data on the impact of oxidation on DNA degradation is inconsistent. The initial study by Booth et al. [28] reported substantial DNA damage caused by oxidation. However, this effect was no longer observed in a follow-up study [40].

Finally, we evaluated a novel combined approach, CAPS+&BS, which is identical to CAPS+, except that pic-borane reduction is replaced by bisulfite conversion. Based on the hypothesis of Xu et al. that 5caC may be a better substrate for bisulfite conversion than 5fC, potentially allowing shorter incubation times [35], this strategy appeared promising. However, in practice, CAPS+&BS showed poor repeatability, particularly when applied to hippocampal DNA, and was associated with severe DNA loss, limiting its applicability.

Recent developments in single-molecule sequencing, such as nanopore-based approaches, enable genome-wide detection of 5hmC without the need for chemical conversion. These methods offer the potential for direct, single-molecule resolution and broader genomic coverage [44]. However, they currently require substantial amounts of high-quality DNA, advanced bioinformatic processing, and do not always provide single-CpG quantification with the same precision as locus-specific methods.

## Conclusions

In this study, we systematically compared bisulfite-based, bisulfite-free, and combined DNA conversion strategies for quantitative 5hmC analysis at single-CpG resolution using pyrosequencing. Our results demonstrate that no single approach is universally optimal; rather, each method exhibits distinct strengths and limitations in terms of accuracy, sensitivity, repeatability, and DNA recovery.

CAPS provided the highest accuracy for quantitative 5hmC determination under controlled conditions using synthetic oligonucleotides. However, its higher false-positive rates resulted in elevated lowest detectable and lowest quantifiable 5hmC levels, limiting its applicability for samples with low 5hmC abundance. In contrast, bisulfite-based protocols, particularly oxidative bisulfite conversion with optimized incubation times, offered superior sensitivity and robustness in genomic DNA samples, despite lower apparent 5hmC levels.

From a practical perspective, bisulfite-based approaches may be advantageous for many laboratories, as conventional bisulfite conversion is well established, widely available, and routinely applied in DNA methylation analysis workflows. Careful optimization of bisulfite incubation conditions is, however, essential to balance conversion efficiency, quantitative accuracy, and DNA preservation.

Overall, our findings highlight the importance of selecting 5hmC quantification strategies based on the biological question, expected 5hmC abundance, and practical constraints, rather than relying on a single method as a general solution.

## Supplementary Information

Below is the link to the electronic supplementary material.

Supplementary Material 1.

## Data Availability

The data supporting the findings of this study are available within the article.

## Abbreviations

CpG: CpG dinucleotide
MGMT: O6-methylguanine-DNA methyltransferase
PCR: Polymerase chain reaction
PSQ: Pyrosequencing
SD: Standard deviation
LDL: Lowest detectable level
LQL: Lowest quantifiable level
C: Cytosine
5mC: 5-methylcytosine
5hmC: 5-hydroxymethylcatosine
5fC: 5-formylcytosine
5caC: 5-carboxylcytosine
