# Supplementary material for: Comparative validation of oxidative bisulfite sequencing (oxBS) and chemical-assisted pyridine borane sequencing (CAPS) protocols for locus-specific 5-hydroxymethylcytosine quantification
Source: Epigenetics Chromatin. 2026 Apr 20;19:29. doi: 10.1186/s13072-026-00672-3 (PMC13352651; doi:10.1186/s13072-026-00672-3)
Supplement: Supplementary file 1 — Supplementary Material 1. [file 13072_2026_672_MOESM1_ESM.docx]

Comparative validation of oxidative bisulfite sequencing (oxBS) and chemical-assisted pyridine borane sequencing (CAPS) protocols for locus-specific 5-hydroxymethylcytosine quantification

Katharina Pühringer ^1,2^, Philipp Czarda ^1^, Sebastian Iluca ^1^, Benno Fehringer ^1^, Pece Sherovski ^3^, Angelica Ohindovschi ^4^, Andreas Hainfellner ^5^, Lukas Reissig ^5^, Wolfgang Weninger ^5^, and Margit Cichna-Markl ^1,^*

^1^ Institute of Analytical Chemistry, Faculty of Chemistry, University of Vienna, Vienna, Austria

^2^ Vienna Doctoral School in Chemistry (DoSChem), University of Vienna, Vienna, Austria

^3^ Institute of Chemistry, Faculty of Natural Sciences and Mathematics, Ss. Cyril and Methodius University in Skopje, Skopje, North Macedonia

^4^ Department of Pharmacognosy and Pharmaceutical Botany, Nicolae Testemițanu State University of Medicine and Pharmacy, Chișinău, Republic of Moldova

^5^ Division of Anatomy, Centre for Anatomy and Cell Biology, Medical University of Vienna, Vienna, Austria

***** Correspondence: [margit.cichna@univie.ac.at](mailto:margit.cichna@univie.ac.at) (MCM)

R-packages:

Wickham et al. (2019). Welcome to the tidyverse. Journal of Open Source Software, 4(43), 1686. <https://doi.org/10.21105/joss.01686>.

Hadley Wickham (2016). ggplot2: Elegant Graphics for Data Analysis. Springer-Verlag New York. <https://ggplot2.tidyverse.org>

Baptiste Auguie (2017). gridExtra: Miscellaneous functions for “grid” graphics (Version 2.3). <https://CRAN.R-project.org/package=gridExtra>

David Robinson, Alex Hayes and Simon Couch (2022). broom: Convert Statistical Objects into Tidy Tibbles. R package version 1.0.6. <https://CRAN.R-project.org/package=broom>

Mike FC, Trevor L. Davis, ggplot2 authors (2025). ggpattern: ggplot2 Pattern Geoms. R package version 1.2.1. <https://CRAN.R-project.org/package=ggpattern>

Jeroen Ooms (2025). writexl: Export Data Frames to Excel ‘xlsx’ Format. R package version 1.5.4. <https://CRAN.R-project.org/package=writexl>
